# Effectiveness of successive booster vaccine doses against SARS-CoV-2 related mortality in residents of long-term care facilities in the VIVALDI study

**DOI:** 10.1093/ageing/afad141

**Published:** 2023-08-12

**Authors:** Oliver Stirrup, Madhumita Shrotri, Natalie L Adams, Maria Krutikov, Borscha Azmi, Igor Monakhov, Gokhan Tut, Paul Moss, Andrew Hayward, Andrew Copas, Laura Shallcross

**Affiliations:** Institute for Global Health, University College London, London, UK; UCL Institute of Health Informatics, University College London, London, UK; UCL Institute of Health Informatics, University College London, London, UK; UCL Institute of Health Informatics, University College London, London, UK; UCL Institute of Health Informatics, University College London, London, UK; UK Health Security Agency, London, UK; Institute of Immunology and Immunotherapy, University of Birmingham, Birmingham, UK; Institute of Immunology and Immunotherapy, University of Birmingham, Birmingham, UK; UCL Institute of Epidemiology & Healthcare, University College London, London, UK; Health Data Research UK, London, UK; Institute for Global Health, University College London, London, UK; UCL Institute of Health Informatics, University College London, London, UK

**Keywords:** SARS-CoV-2, COVID-19, omicron, vaccine effectiveness, long-term care facilities, older people

## Abstract

**Background:**

Severe acute respiratory syndrome coronavirus 2 (SARS-CoV-2) caused severe disease in unvaccinated long-term care facility (LTCF) residents. Initial booster vaccination following primary vaccination is known to provide strong short-term protection, but data are limited on duration of protection and the protective effect of further booster vaccinations.

**Objective:**

To evaluate the effectiveness of third, fourth and fifth dose booster vaccination against SARS-CoV-2 related mortality amongst older residents of LTCFs.

**Design:**

Prospective cohort study.

**Setting:**

LTCFs for older people in England participating in the VIVALDI study.

**Methods:**

Residents aged >65 years at participating LTCFs were eligible for inclusion if they had at least one polymerase chain reaction or lateral flow device result within the analysis period 1 January 2022 to 31 December 2022. We excluded individuals who had not received at least two vaccine doses before the analysis period. Cox regression was used to estimate relative hazards of SARS-CoV-2 related mortality following 1–3 booster vaccinations compared with primary vaccination, stratified by previous SARS-CoV-2 infection and adjusting for age, sex and LTCF size (total beds).

**Results:**

A total of 13,407 residents were included. Our results indicate that third, fourth and fifth dose booster vaccination provide additional short-term protection against SARS-CoV-2 related mortality relative to primary vaccination, with consistent stabilisation beyond 112 days to 45–75% reduction in risk relative to primary vaccination.

**Conclusions:**

Successive booster vaccination doses provide additional short-term protection against SARS-CoV-2 related mortality amongst older LTCF residents. However, we did not find evidence of a longer-term reduction in risk beyond that provided by initial booster vaccination.

## Key Points

- Third, fourth and fifth dose booster vaccination provide additional short-term protection against SARS-CoV-2 related mortality relative to primary vaccination amongst older residents of long-term care facilities.
- There was waning in the level of protection following third, fourth and fifth vaccine doses.
- We did not find evidence of sustained benefit from fourth or fifth dose vaccination relative to the protection provided by the first three doses.

## Introduction

Long-term care facilities (LTCFs) in the UK were severely impacted by COVID-19 early in the pandemic [1]. As such, LTCF staff and residents were prioritised for primary vaccination against syndrome coronavirus 2 (SARS-CoV-2) starting in December 2020 [2] and for additional booster vaccinations.

We previously reported waning of protection against infection and severe outcomes following primary vaccination in LTCF residents from 84 days following second dose [3], with improvement in protection against severe outcomes observed following first booster vaccine dose [3] that remained after emergence of the Omicron variant [4]. The present study focuses on mortality because changes to UK testing policy in LTCFs within the period analysed make it challenging to analyse effects on SARS-CoV-2 infection incidence or evaluate hospital admissions. We aimed to produce an updated evaluation of the effectiveness of third, fourth and fifth dose booster vaccination against SARS-CoV-2 associated death amongst residents of LTCFs in England in 2022.

## Methods

VIVALDI is a prospective cohort study investigating SARS-CoV-2, including residents and staff of LTCFs providing residential and/or nursing care for older people in England [5]. Following national guidelines, residents underwent monthly routine polymerase chain reaction (PCR) testing until end of March 2022, when policy switched to symptomatic and outbreak testing only. Residents of participating LTCFs aged >65 years were eligible for inclusion if they had at least one PCR or lateral flow device (LFD) result recorded within the analysis period 1 January 2022 to 31 December 2022. We excluded individuals who had not received at least two vaccine doses before the analysis period [4] because unvaccinated residents are substantially more likely to be receiving end of life care. Individuals with third vaccine dose recorded before 14 September 2021, fourth dose before 21 March 2022 and fifth dose before 5 September 2022 were excluded as these dates corresponded to national roll-out to residents. Individuals whose first test record was a positive result within 2022 were excluded, as they would have only been considered as under follow-up from time of infection. The analysis period was chosen to allow our prior estimates of vaccine effectiveness to be updated whilst retaining a period when asymptomatic testing was still in use in order to ascertain the cohort.

As previously we retrieved all available PCR and LFD results from the national testing programme through the COVID-19 Datastore. Test results and vaccination (National Immunisation Management Service) and mortality (Office for National Statistics) data from national records were linked to study participants using pseudo-identifiers based on National Health Service (NHS) numbers [3]. COVID-19 death was defined as death within 28 days of positive PCR or LFD test or with COVID-19 recorded as primary or secondary cause of death on the death certificate. The legal basis to access data is provided by Health Research Authority Confidentiality Advisory Group approval (21/CAG/0156). Ethical approval was obtained from South Central-Hampshire B Research Ethics Committee (20/SC/0238). SARS-CoV2 serological test results for IgG antibodies to nucleocapsid protein (ARCHITECT system (Abbott, Maidenhead, UK)) were linked in a subset of participants who consented to blood sampling specifically for the VIVALDI study [6].

We used Cox regression models to derive adjusted hazard ratios (HRs) for risk of SARS-CoV-2 linked death. Vaccination status was included as time-varying covariable, with reference category two vaccine doses and categorical exposure groups following doses 3–5 (full details Appendix S1: Further details of statistical analysis). Baseline hazard was defined over calendar time. Analysis was stratified by evidence of SARS-CoV-2 infection prior to risk period, based on combined PCR and LFD results, hospital admission records and nucleocapsid antibody results where available. We adjusted for sex (binary variable), age (five-knot restricted cubic spline term) and LTCF size (number of beds, linear term).

## Results

Analysis included 13,407 residents from 327 LTCFs (Figure S1), of which 3,411 (25.4%) had recorded evidence of SARS-CoV-2 infection prior to the analysis period. Median age was 86.6 years (interquartile range, 80.2–91.8) and 4,132 residents died during the analysis period, of which 428 were associated with SARS-CoV-2.

**Figure 1:**
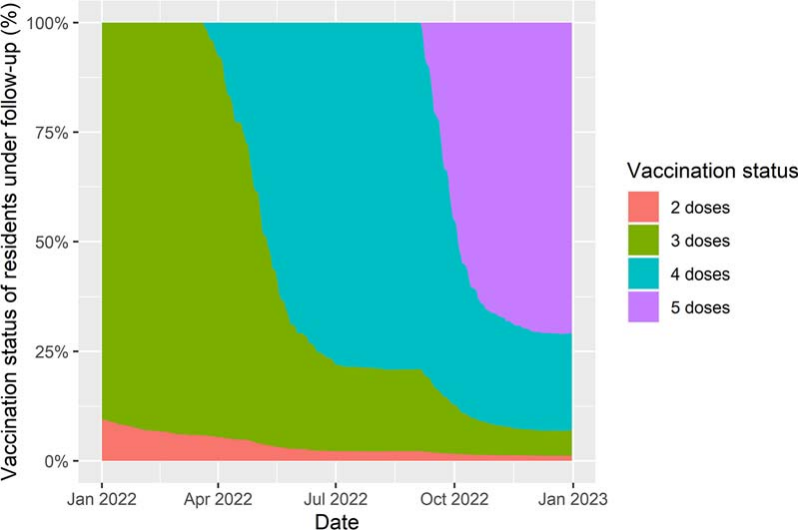
Plot of booster vaccination status of residents of long-term care facilities included in the analysis. The percentage of residents with each number of vaccine doses is given amongst those in follow-up for the analysis at any given point in time.

The majority of residents had received AstraZeneca for first (*n* = 8,206, 61.2%) and second (*n* = 8,372, 62.4%) vaccine doses, with Pfizer used in nearly all other cases (*n* = 5,198 and *n* = 5,031, respectively). Third-dose booster vaccination had been received by 12,072 (90.0%) residents prior to analysis period, and 13,104 (97.7%) by the end (98.8% of survivors to year-end) (Figure 1). First booster doses were Pfizer in the majority (*n* = 12,474, 95.2%), with Moderna also used (*n* = 593, 4.5%). Second boosters (fourth dose) had been received by 10,846 (80.9%, 93.0% of survivors) and third boosters (fifth dose) by 7,311 (54.5%, 70.8% of survivors) of residents by end of analysis period. The majority were Moderna vaccines for second booster (6,421 (59.2%)) and for third booster (5,991 (81.9%)). By end of study period 8,621 (64.3%) residents had received a bivalent vaccine (based on spike protein of BA.1 lineage and Wuhan strain), mostly as third booster dose but in some as first (*n* = 120) or second (*n* = 1,455) booster.

**Figure 2:**
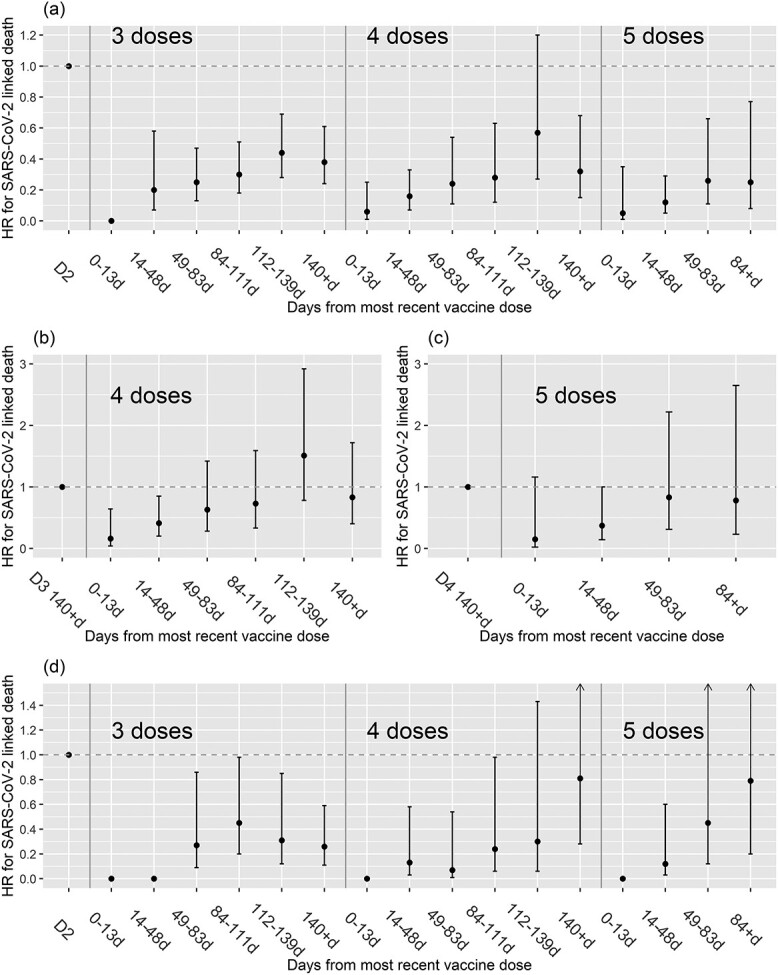
Plot of estimated HR for SARS-CoV-2 linked death (within 28 days of a positive PCR or LFD test, and/or recorded on death certificate) following receipt of booster vaccine doses, amongst long-term care facility residents without evidence of prior SARS-CoV-2 infection (a)–(c) and in those with prior SARS-CoV-2 infection (d). Results are shown for 3–5 doses relative to two-dose vaccination in (a) and (d), for four doses relative to three doses beyond 140 days in (b) and for five doses relative to four doses beyond 140 days in (c).

In residents without known prior SARS-CoV-2 infection, first booster reduced risk of SARS-CoV-2 linked death after 0–13 days (none observed), 14–48 days (HR 0.20, 0.07–0.58), 49–83 days (0.25, 0.13–0.47) and 84–111 days (0.30, 0.18–0.51), with some waning in level of protection by 112–139 days (0.44, 0.28–0.69) and 140+ days (0.38, 0.24–0.61) (Figure 2, Table S1). A similar pattern was observed following fourth and fifth dose vaccination, although confidence intervals for HRs were wider for fourth dose beyond 84 days and for fifth dose (Figure 2d) because of lower available follow-up time and lower incidence of SARS-CoV-2 infection in the latter half of 2022. Residents with known infection prior to analysis period were at reduced risk of death relative to those without prior infection (0.55,0.28–1.08; amongst those with two-dose vaccination). Within this group, the pattern of further protection from booster vaccination was similar to those without known prior infection, although there is greater uncertainty in estimates. Adding a parameter to the model representing receipt of a bivalent vaccine indicated possible greater protection but with wide confidence interval (0.81, 0.29–2.25).

## Discussion

We found evidence that third, fourth and fifth dose booster vaccination provide additional short-term protection against SARS-CoV-2 linked mortality amongst LTCF residents, relative to primary vaccination. The pattern of waning of protection appeared to be similar for successive booster doses, stabilising beyond 112 days at 45–75% reduction in risk relative to primary vaccination.

Our findings are consistent with data on fourth dose vaccination of LTCF residents in USA [7] and Canada [8] showing additional short-term protection against SARS-CoV-2 related death. Grewal *et al.* also found that the additional protection against severe outcomes from fourth relative to third dose waned over time, with negligible protection from 168 days [9]. We are not aware of any publications on fifth dose vaccination of LTCF residents, but fifth dose has been estimated to provide 46.4% additional protection against hospitalisation in the following 3 months compared to four doses in the ≥75y age group in the UK [10].

Our data suggest that the combination of booster vaccination with prior SARS-CoV-2 infection provides particularly strong protection against subsequent mortality from the virus, in line with previous studies in the general population [11]. We are likely to have underestimated the protective effect of prior infection, as we have previously found the cumulative incidence of detected SARS-CoV-2 infection to be substantially higher amongst residents who underwent testing for anti-nucleocapsid antibodies [6]. This suggests that many ‘unexposed’ residents in our analyses may have in fact been previously infected, although the prevalence of prior infection may be lower in residents who were admitted to care homes in the second half of 2021 and 2022 compared to those resident since 2020. Underestimation of prior infection is unlikely to have had a substantial impact on our estimates of protection provided by booster vaccination, given that the impact did not appear to differ by prior infection status, if we can assume there was not a strong association between undetected prior infection and booster uptake in the LTCF setting.

A limitation of our study is that we lacked data on co-morbidities or frailty level of individuals included, which may represent unmeasured confounding if associated with booster uptake. Another limitation is that older residents of LTCFs are a frail population with very high background rates of mortality and it is likely that a substantial proportion of SARS-CoV-2 related deaths in our analysis represent deaths ‘with SARS-CoV-2’ rather than directly caused by the virus, although attribution of a single cause of death is often not clear in this context [12].

It is possible that the primarily bivalent fifth dose vaccination provided some additional long-term protection, but the pattern of reduction in risk over time did not differ markedly from that observed for prior booster doses. This is consistent with data from the general population in the USA [13], perhaps due to the fact that different Omicron sub-lineages were circulating by the time that bivalent vaccines based on BA.1 lineage were rolled out [14].

We provide evidence that successive booster doses provide additional short-term protection against SARS-CoV-2 related mortality, but lack of sustained benefit from fourth or fifth dose vaccination relative to initial booster dose. These findings raise important questions for policymakers regarding the likely effectiveness and cost-effectiveness of future rounds of SARS-CoV-2 booster vaccination and highlight the continued need to monitor vaccine effectiveness in this vulnerable population.

## Declaration of Conflicts of Interest

LS reports grants from the Department of Health and Social Care during the conduct of the study and is a member of the Social Care Working Group, which reports to the Scientific Advisory Group for Emergencies. AH reports funding from the COVID Core Studies Programme and is a member of the New and Emerging Respiratory Virus Threats Advisory Group at the Department of Health and Environmental Modelling Group of the Scientific Advisory Group for Emergencies. All other authors declare no competing interests.

## Supplementary Material

aa-23-0426-File002_afad141

## Data Availability

De-identified test results and limited meta-data will be made available for use by researchers in future studies, subject to appropriate research ethical approvals, once the VIVALDI study cohort has been finalised.
