# Supplementary material for: Effectiveness of successive booster vaccine doses against SARS-CoV-2 related mortality in residents of long-term care facilities in the VIVALDI study
Source: Age Ageing. 2023 Aug 12;52(8):afad141. doi: 10.1093/ageing/afad141 (PMC10438206; doi:10.1093/ageing/afad141)
Supplement: aa-23-0426-File002_afad141 [file aa-23-0426-file002_afad141.docx]

*Supplementary Appendix to:*

**Effectiveness of successive booster vaccine doses against SARS-CoV-2 related mortality in residents of Long-Term Care Facilities in the VIVALDI study**

Oliver Stirrup, Madhumita Shrotri, Natalie L. Adams, Maria Krutikov, Borscha Azmi, Igor Monakhov, Gokhan Tut, Paul Moss, Andrew Hayward, Andrew Copas, Laura Shallcross

Contents:

- Further details of statistical analysis (page 2)

- Figure S1: Participant inclusion flow chart (page 3)

- Table S1: Crude event rates and adjusted hazard ratios against SARS-CoV-2 associated death (page 4)

**Further details of statistical analysis**

We used Cox regression models to derive adjusted hazard ratios (HRs) for risk of SARS-CoV-2 linked death, with vaccination status included as a time-varying covariable. The reference category was 2 vaccine doses with ≥84 days elapsed from Dose 2, but this was varied to create graphical summaries of the estimated effect of successive doses. Exposure categories were 0–13, 14–48, 49–83, 84-111, 112-139 and ≥140 days following Dose 3 and Dose 4, and 0–13, 14–48, 49–83 and ≥84 days following Dose 5. Individuals entered the risk period on 1^st^ January 2022, or date of their first recorded PCR/LFD result within VIVALDI if later. Individuals with positive PCR/LFD result within 30 days prior to 1^st^ January 2022 entered the risk period from the 31^st^ day post-positive test. Individuals exited the risk period at the earliest of: death or end of analysis period. Individuals were additionally censored at 29 days post-positive SARS-CoV-2 test if they survived to this point; COVID-related death from the original infection would be possible beyond this time but the individual would also be at lower risk for a new SARS-CoV-2 infection, and so censoring at 29 days was chosen to match our mortality outcome definition. 95% CIs were calculated using robust SEs accounting for dependence of infection events within LTCFs. All statistical analyses were conducted using STATA 17.0.

**Figure S1** Flow chart of inclusion of older long term care facility residents in the analysis

|  |  | Excluded |  |
| --- | --- | --- | --- |
| Total participant records | 72116 |  |  |
|  | **↓** | 9176 | No PCR or LFD results (at any time) |
|  | 62940 |  |  |
|  | **↓** | 263 | Missing demographic data |
|  | 62677 |  |  |
|  | **↓** | 33338 | Resident<65, or staff |
|  | 29339 |  |  |
|  | **↓** | 9258 | Death before analysis start date |
|  | 20081 |  |  |
|  | **↓** | 2874 | Not in Vivaldi home in analysis period |
|  | 17207 |  |  |
|  | **↓** | 1647 | No PCRs or LFDs within analysis period |
|  | 15560 |  |  |
|  | **↓** | 1051 | 2-dose vacc. not complete 83d before start date |
|  | 14509 |  |  |
|  | **↓** | 194 | Booster dose before roll-out |
|  | 14315 |  |  |
|  | **↓** | 904 | Positive on first day of at-risk period |
| Residents included | 13407 |  |  |

**Table S1** Crude event rates and adjusted hazard ratios against SARS-CoV-2 associated death (within 28 days of a positive PCR or LFD test, and/or recorded on death certificate) for LTCF residents, by prior SARS-CoV-2 exposure, and vaccination status

| **SARS-CoV-2 mortality** | | |  |  |  |  |
| --- | --- | --- | --- | --- | --- | --- |
| **Prior SARS-CoV-2 exposure** | **Vaccination status** | **Person-days** | **Deaths** | **IR /1000pd** | **HR (95% CI)*** | **HR (95% CI) *** |
| Unexposed | D2 84+d | 71837 | 35 | 0.49 | Ref. |  |
|  | D3 0-13d | 6471 | 0 | 0 | 0.00 (0.00-0.00) |  |
|  | D3 14-48d | 39206 | 4 | 0.1 | 0.20 (0.07-0.58) |  |
|  | D3 49-83d | 115192 | 15 | 0.13 | 0.25 (0.13-0.47) |  |
|  | D3 84-111d | 193274 | 39 | 0.2 | 0.30 (0.18-0.51) |  |
|  | D3 112-139d | 189472 | 50 | 0.26 | 0.44 (0.28-0.69) |  |
|  | D3 140+d | 432958 | 81 | 0.19 | 0.38 (0.24-0.61) |  |
|  | D4 0-13d | 66886 | 2 | 0.03 | 0.06 (0.01-0.25) |  |
|  | D4 14-48d | 176815 | 10 | 0.06 | 0.16 (0.07-0.33) |  |
|  | D4 49-83d | 168385 | 15 | 0.09 | 0.24 (0.11-0.54) |  |
|  | D4 84-111d | 119450 | 14 | 0.12 | 0.28 (0.12-0.63) |  |
|  | D4 112-139d | 92701 | 16 | 0.17 | 0.57 (0.27-1.20) |  |
|  | D4 140+d | 90740 | 11 | 0.12 | 0.32 (0.15-0.68) |  |
|  | D5 0-13d | 43722 | 1 | 0.02 | 0.05 (0.01-0.35) |  |
|  | D5 14-48d | 114653 | 8 | 0.07 | 0.12 (0.05-0.29) |  |
|  | D5 49-83d | 99414 | 10 | 0.1 | 0.26 (0.11-0.66) |  |
|  | D5 84+d | 41549 | 5 | 0.12 | 0.25 (0.08-0.77) |  |
| Exposed | D2 84+d | 40015 | 11 | 0.27 | 0.55 (0.28-1.08) | Ref. |
|  | D3 0-13d | 3932 | 0 | 0 |  | — |
|  | D3 14-48d | 20433 | 0 | 0 |  | — |
|  | D3 49-83d | 51452 | 4 | 0.08 |  | 0.27 (0.09-0.86) |
|  | D3 84-111d | 81134 | 13 | 0.16 |  | 0.45 (0.20-0.98) |
|  | D3 112-139d | 78516 | 8 | 0.1 |  | 0.31 (0.12-0.85) |
|  | D3 140+d | 174497 | 12 | 0.07 |  | 0.26 (0.11-0.59) |
|  | D4 0-13d | 29654 | 0 | 0 |  | — |
|  | D4 14-48d | 78026 | 2 | 0.03 |  | 0.13 (0.03-0.58) |
|  | D4 49-83d | 74409 | 1 | 0.01 |  | 0.07 (0.01-0.54) |
|  | D4 84-111d | 52959 | 3 | 0.06 |  | 0.24 (0.06-0.98) |
|  | D4 112-139d | 40544 | 2 | 0.05 |  | 0.30 (0.06-1.43) |
|  | D4 140+d | 38498 | 6 | 0.16 |  | 0.81 (0.28-2.33) |
|  | D5 0-13d | 19276 | 0 | 0 |  | — |
|  | D5 14-48d | 49902 | 2 | 0.04 |  | 0.12 (0.03-0.60) |
|  | D5 49-83d | 43712 | 4 | 0.09 |  | 0.45 (0.12-1.65) |
|  | D5 84+d | 19395 | 4 | 0.21 |  | 0.79 (0.20-3.07) |

*HR values in the two columns are from mathematically identical statistical models, but HRs in right-hand column are expressed relative to ‘D2 84+d’ vaccine status in individuals with prior SARS-CoV-2 infection.
